# Baicalin Represses C/EBP*β* via Its Antioxidative Effect in Parkinson's Disease

**DOI:** 10.1155/2020/8951907

**Published:** 2020-05-21

**Authors:** Kecheng Lei, Yijue Shen, Yijing He, Liwen Zhang, Jingxing Zhang, Weifang Tong, Yichun Xu, Lingjing Jin

**Affiliations:** ^1^Neurotoxin Research Center of Key Laboratory of Spine and Spinal Cord Injury Repair and Regeneration of Ministry of Education, Neurological Department of Tongji Hospital, Tongji University School of Medicine, 200065 Shanghai, China; ^2^Department of Pathology and Laboratory Medicine, Emory University School of Medicine, 30322 Atlanta, Georgia, USA; ^3^National Engineering Research Center for Biochip, Shanghai Biochip Limited Corporation, Shanghai, China; ^4^Institute of Digestive Diseases, School of Medicine, Tongji University, China

## Abstract

Parkinson's disease (PD) is a neurodegenerative disease characterized by the gradual loss of dopaminergic (DA) neurons in the substantia nigra (SN) and the formation of intracellular Lewy bodies (LB) in the brain, which aggregates *α*-synuclein (*α*-Syn) as the main component. The interest of flavonoids as potential neuroprotective agents is increasing due to its high efficiency and low side effects. Baicalin is one of the flavonoid compounds, which is a predominant flavonoid isolated from Scutellaria baicalensis Georgi. However, the key molecular mechanism by which Baicalin can prevent the PD pathogenesis remains unclear. In this study, we used bioinformatic assessment including Gene Ontology (GO) to elucidate the correlation between oxidative stress and PD pathogenesis. RNA-Seq methods were used to examine the global expression profiles of noncoding RNAs and found that C/EBP*β* expression was upregulated in PD patients compared with healthy controls. Interestingly, Baicalin could protect DA neurons against reactive oxygen species (ROS) and decreased C/EBP*β* and *α*-synuclein expression in pLVX-Tet3G-*α*-synuclein SH-SY5Y cells. In a 1-methyl-4-phenyl-1,2,3,6-tetrahydropyridine (MPTP) induced PD mouse model, the results revealed that treatment with Baicalin improved the PD model's behavioral performance and reduced dopaminergic neuron loss in the substantia nigra, associated with the inactivation of proinflammatory cytokines and oxidative stress. Hence, our study supported that Baicalin repressed C/EBP*β* via redox homeostasis, which may be an effective potential treatment for PD.

## 1. Background

Oxidative stress has been implicated as a key contributor to the progression of Parkinson's disease (PD) [1]. Because of the presence of enzymes such as tyrosine hydroxylase (TH) and monoamine oxidase (MAO), the neurotransmitter dopamine can be a major source of oxidative stress [2]. Although the human brain comprises less than 5% of total body weight, over 20% of the whole body's total oxygen is supplied to it, with part of oxygen subsequently converted into reactive oxygen species (ROS) [3]. Oxidative stress is considered as the common underlying source that leads to cellular dysfunction and demise, the idiopathic and genetic causes of PD [3, 4]. Overexpression of oxidative stress may lead to excitotoxicity, mitochondrial dysfunction, protein misfolding and aggregation, and cellular apoptosis, which are all *in vitro* indicators of PD [5]. It is also believed that the increased levels of oxidized lipids are the common underlying mechanism that leads to dopaminergic neuronal loss in the substantia nigra (SN) and motor dysfunctions in PD patients [6].

*α*-Synuclein accumulates in Lewy bodies, which is the hallmark in PD pathology and leads to neurodegeneration and the progression of the clinical symptoms [7]. Although its exact role in neuropathology is unclear, evidence suggests that overexpression of *α*-synuclein might lead to oxidative stress [8] and neuroinflammation [9]. Then, oxidative stress can modulate the *α*-synuclein structure, leading to other formations of the protein, including fibrils and oligomers [10], the latter of which can develop into positive regulation of ROS. The phosphorylation of *α*-synuclein at Ser-129, the major phosphorylation site, has been demonstrated from an animal model study to produce neurotoxic effects [11]. Moreover, oxidative stress leading to ROS production and *α*-synuclein aggregation is one of the proposed mechanisms for the death of dopaminergic neurons in PD patients [12].

It is also notable that the mitochondrial complex I damage is demonstrated to be one of the primary PD pathological animal models due to the administration of mitochondrial toxins, such as MPTP and rotenone, leading to the formation of *α*-synuclein aggregates and oxidative stress [13]. Methyl-4-phenyl-1,2,3,6-tetrahydropyridine (MPTP), a mitochondrial complex I inhibitor, is metabolized into the toxin 1-methyl-4-phenylpyridine (MPP^+^) by monoamine oxidase B (MAO-B) and later taken up by dopaminergic neurons, which finally lead to neuronal death and ROS production [14]. In rats, chronic administration of rotenone caused selective nigral dopaminergic neuron loss and a significant reduction in complex I activity while at the same time, the ROS level increased [15]. Altogether, these findings suggest that mitochondrial respiratory chain impairment, in particular, complex I deficiency, and the subsequent increase in ROS production may directly contribute to the pathology of PD.

Usually, it is mitochondria's ability to produce ATP appropriately in response to energy demands [16]. Meanwhile, the transcription factor CCAAT/enhancer-binding protein beta (C/EBP*β*), which is expressed in the brain, is also involved in the regulation of ATP synthesis [17]. Remarkably, C/EBP*β*−/− mice exhibit resistance to excitotoxicity-induced neuronal cell death, which indicates that C/EBP*β* might regulate gene expression implicated in brain damage [18].

There have been many reports discussing effective antioxidant treatment for PD, as well as conventional compounds that possessed antioxidant activity [19, 20]. Therefore, it is reasonable to suggest that targeting oxidative stress may be an effective strategy for PD medicine. Natural compounds have always been attractive targets for discovering new drug candidates, and many flavonoid derivatives are effective in preventing oxidative stress [21]. For instance, Hesperidin, the main flavanone derivative of citrus fruits, can alleviate cognitive impairment and oxidative stress in a mouse model of Alzheimer's disease [22]. Similarly, myricitrin, a flavonoid isolated from Chinese bayberry bark and fruit, demonstrated a protective effect on MPP+ induced mitochondrial dysfunction in a DJ-1-dependent manner in SN4741 cells [23]. Taking into account information about flavonoids, the focus of our paper is to discuss Baicalin, which is also the flavonoid derivatives, the principal component in the roots of Scutellaria radix, known as Huang Qin in Chinese traditional medicine [21, 24]. In recent years, several studies have shown that Baicalin displays a potent neuroprotective effect in various *in vitro* and *in vivo* models of neuronal injuries [25]. In particular, Baicalin effectively prevents neurodegenerative diseases through various pharmacological mechanisms, including antiexcitotoxicity, antiapoptosis, and anti-inflammation, promoting the expression of neuronal protective factors [26]. However, the mechanism of which Baicalin can inhibit neurodegeneration and regulate redox homeostasis is unclear. In this study, we used RNA-Seq to examine the global expression profiles of noncoding RNAs in PD patients and healthy controls, and then, we demonstrated that Baicalin could protect cells from neurotoxicity *in vitro* and *in vivo*. Subsequently, Gene Ontology analysis displayed that, compared with the healthy controls, many processes overrepresented in PD patients were related to glucocorticoid receptor binding and cellular response to oxidative stress. Our study may help extend understanding of the roles of oxidative stress and provide new research directions for PD.

## 2. Materials and Methods

### 2.1. Cell Lines and Cell Culture

Human cell line pLVX-Tet3G-*α*-synuclein SH-SY5Y was provided by Dr. Jingxing Zhang and supplemented with G418 (100 *μ*g/ml). The cells were cultured in 1640 medium (Life Technologies, USA), supplemented with 10% fetal bovine serum (Hyclone, USA), penicillin (100 U/ml), and streptomycin (100 U/ml) (ABAM Life Technologies, California, USA). Cell cultures were maintained in 5% CO_2_ and air humidified in a 37°C incubator [27].

### 2.2. Chemicals and Reagents

Baicalin was purchased from the company (Sigma, USA), and the stock solutions were prepared in dimethyl sulfoxide (DMSO) (Sigma, USA). For *in vivo* experiments, Baicalin was dissolved in sterile PBS.

### 2.3. Bioinformatic Analysis

Differentially expressed genes (DEGs) were determined from a treated versus control comparison of log2-transformed expression measurements using the R package (http://www.bioconductor.org/packages/release/bioc/html/edgeR.html), and the resulting *p* values were adjusted using Benjamini and Hochberg's approach for controlling the false discovery rate (FDR) [28]. Differentially expressed genes (DEGs) with statistical significance were identified through volcano plot filtering. The thresholds for DEG were absolute log2 fold change > 1 and *p* value < 0.01. Hierarchical clustering was performed using pheatmap package in R. To understand the potential biological functions of DEGs, we used clusterProfiler on R platform (https://bioconductor.org/packages/release/bioc/html/clusterProfiler.html). GO terms with corrected *p* value less than 0.05 were considered significantly enriched by DEGs.

### 2.4. Quantitative Real-Time PCR

Total RNA was extracted using a TRIzol reagent (Invitrogen, California, USA) according to the manufacturer's instructions. Reverse transcription was performed with SuperScript III reverse transcriptase (Life Technologies, #18080085), and primers were designed and purchased from TaqMan: CEBPB (Hs00270923_s1), Cebpb (Mm00843434_s1), SNCA (Hs00240906_m1), Snca (Mm01188700_m1), Il1b (Mm00434228_m1), Il6 (Mm00446190_m1), TNF-*α* (Mm00443258_m1), and TGF*β* (Mm01178820_m1). Real-time PCR was performed with a TaqMan Universal Master Mix Kit (Life Technologies, #4304473) by ABI 7500 Fast Real-Time PCR System. The relative quantification of the target genes was calculated by the comparative cycle threshold (CT) (2^−*ΔΔ*CT^) method. The expression of GAPDH was used as an endogenous control. The relative quantification of gene expression was calculated by the 2-^*ΔΔ*CT^ method. All tests were performed in triplicate [29].

### 2.5. Protein Extraction and Western Blot Analysis

After the cell treatment under different conditions, the cells were harvested and the total proteins were extracted. Equal amounts of the proteins were loaded on SDS-PAGE gels, and the western blot assays were performed as previously described. *α*-Synuclein (#610787, BD Biosciences, USA), *α*-synuclein pS-129 (ab51253, Abcam, USA), p-C/EBP*β* (#3084, CST, USA), C/EBP*β* (#7962, Santa Cruz, USA), TH (#2792 CST, USA), and cleaved caspase-3 (#9664, CST, USA) were used at a final concentration of 1 mg/ml and were incubated overnight at 4°C in the presence of 5% nonfat milk powder. *β*-Actin (#3700, CST, USA) was used as the loading control [30].

### 2.6. Cell Viability Assay

Cell cytotoxicity was assessed in vitro using the 3-(4,5-dimethylthiazol-2-yl)-2,5-diphenyltetrazolium bromide (MTT) assay. After different treatments, 20 *μ*l of MTT (5 mg/ml in PBS, Sigma, USA) was added to each well and the plates were incubated for 2 h. The resulting formazan product was dissolved with DMSO, and the absorbance at a wavelength of 490 nm was read using a microplate reader (BioTek Instruments Inc., USA) [31]. All tests were performed in triplicate.

### 2.7. Intracellular ROS Measurement

The level of intracellular reactive oxygen species (ROS) was detected by the DCFH-DA method. After different treatments, cells were collected and then incubated with 10 *μ*M DCFH-DA (ROS dye, #C6827, Invitrogen, USA) for 1 hour at 37°C. The fluorescence intensity was measured by a microplate reader (BioTek Instruments Inc., USA) with settings at excitation and emission equal to 485/535 nm, and all tests were performed in triplicate [32].

### 2.8. Lactate Dehydrogenase (LDH) Cytotoxicity Assay

The level of LDH was detected by LDH assay kits (Promega Corporation, USA). After different treatments, the 100 *μ*l cell medium of each sample was collected and incubated with 50 *μ*l of the CytoTox 96® Reagent for 30 minutes at room temperature. Then, 50 *μ*l of Stop Solution was added to each well, and the absorbance at 490 nm was recorded. All tests were performed in triplicate.

### 2.9. Protein Carbonyl Assay Measurements and GSH/GSSG Ratio

After different treatments, the protein carbonyl level and GSH/GSSG ratio were measured from cell homogenates using a Protein Carbonyl Assay Kit (#ab126287, Abcam, Cambridge, MA, USA) and GSH/GSSG-Glo™ (Promega Corporation, USA), respectively, according to the manufacturer's guidelines [33]. All tests were performed in triplicate.

### 2.10. JC-1 Mitochondrial Membrane Potential Assay

After different treatments, the cells were washed by PBS and stained with 10 *μ*M JC-1 (Cayman, USA) for 1 hour at 37°C. Finally, the cells were photographed with a fluorescence microscope (Nikon, Japan) at Ex488 nm/Em535 nm and Ex 540 mm/Em570 nm [34].

### 2.11. *In Vivo* Mouse Model Experiments

Male C57BL/6 mice (weighing 20–30 g) were purchased from Shanghai SLAC Laboratory Animal, housed, and maintained at constant temperature and humidity with a 12 h light/dark cycle in Tongji University. Three-month-old mice (8 per group) were injected a daily i.p. injection of MPTP (30 mg/kg) or saline treatment for 5 days [35] and then i.p. injection with 20 mg/kg and 40 mg/kg Baicalin for 2 weeks. Motor impairments were tested with rotarod tests and grid tests after Baicalin treatment (8 mice per group). In the rotarod tests, mice were trained for 2 min at a speed of 4 r.p.m. and then performed eight trials for a maximum of 5 min with increasing speed starting from 4 r.p.m. to 40 r.p.m. The fall-off time was recorded. For inverted grid tests, mice were placed in the center of a 30 × 30 cm screen with 1 cm wide mesh. The screen was inverted head-over-tail and placed on supports 40 cm above an open cage with deep bedding. Mice were timed until they released their grip or remained for 60 s.

### 2.12. Tissue Preparation

After 2 weeks of treatment and behavior test, mice (3 per group) intended for immunofluorescence (IF) staining and immunohistochemistry (IHC) analysis were euthanasia and transcardially perfused with PBS followed by 4% paraformaldehyde (PFA) in PBS. Brains were postfixed for 24 h in 4% PFA at 4°C and transferred to a solution of 30% sucrose in PBS for 24 h at 4°C. The coronal section of SN and STR was sectioned as 30 *μ*m free-floating sections on a cryostat (Leica CM3050) and kept in PBS at 4°C [36]. The mouse brains intended for cell lysis (2 per group) and mRNA (3 per group) were transcardially perfused with ice-cold PBS and later performed western blotting and quantitative real-time PCR individually.

### 2.13. Immunofluorescence Staining

The number of TH- and GFAP-positive cells in the substantia nigra was estimated using a random sampling stereological counting method. Images were sampled from at least four different points within each substantia nigra section [37]. TH (ab6211), GFAP (ab7260), and DAPI (ab104139) were from Abcam, USA. All immunoreactive cells were counted regardless of the intensity of labeling. The slides were photographed with a fluorescence microscope (Nikon, Japan).

### 2.14. Immunohistochemistry Staining

Sections were prepared, and the expression of 4-HNE in the striatum and substantia nigra was assessed using a technique that has been reported previously [38]. In brief, the endogenous peroxidase activity was inactivated with 10% methanol and 3% hydrogen peroxide (H_2_O_2_) solution in PBS, pH 7.4, for 10 min, and nonspecific binding was blocked with 10% normal serum in TBS containing 0.5% Triton X-100. The sections were incubated overnight at 4°C with anti-4-HNE antibody (1 : 200 dilution in 1% normal serum in PBS containing 0.5% Triton X-100; ab46545, Abcam). The sections were then incubated with HRP-conjugated secondary antibody for 1 h. The slides were identified following DAB incubation for 10 min at room temperature. Finally, photographs were taken using a microscope (Nikon, Japan).

### 2.15. Statistical Analysis

Data visualization and analysis were performed with GraphPad Prism 6 (GraphPad Software Inc., La Jolla, CA, USA). Statistical analysis was performed using either Student's *t*-test or one-way ANOVA. Significant difference among groups was assessed as ^∗^*p* < 0.05, ^∗∗^*p* < 0.01, and ^∗∗∗^*p* < 0.001.

## 3. Results

### 3.1. The Functional Annotation of Bioinformatic Analysis in PD Patients

**Figure 1:**
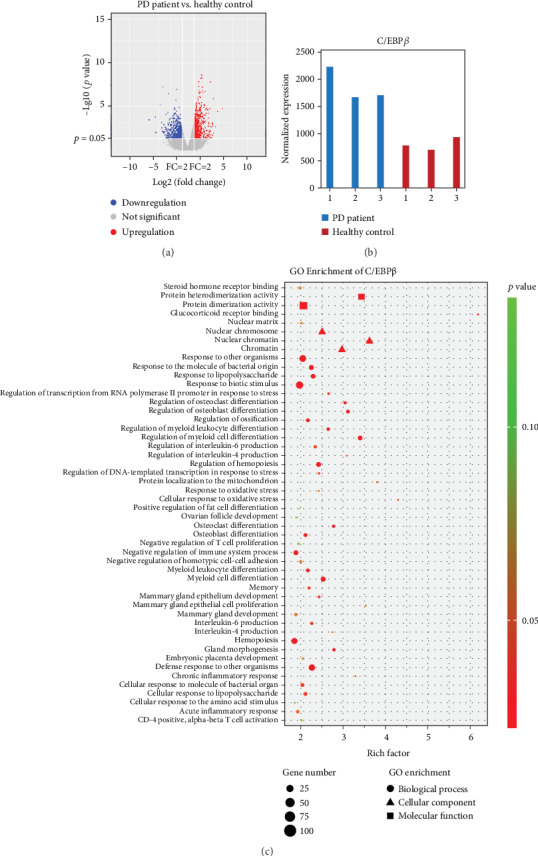
Expression profiles and Gene Ontology (GO) terms for differentially expressed mRNAs between PD patients and healthy control blood samples. (a) Volcano analysis exhibited differentially expressed mRNAs. Blue dots illustrated downregulated genes, and red dots illustrated upregulated genes. (b) C/EBP*β* was upregulated in PD patient samples compared to healthy control samples. (c) GO enrichment analysis for biological processes, cellular component, and molecular function in the interaction networks of C/EBP*β*.

To profile differentially expressed mRNAs of PD patients and healthy controls, we obtained blood samples from both groups. Then, the whole-genome sequencing and alteration were analyzed. The mRNA was upregulated or downregulated, respectively, by more than twofold in PD patients vs. healthy controls (*p* < 0.05) (Figure 1(a)). Among them, the results showed that C/EBP*β* was upregulated in PD patient blood samples compared with healthy controls (Figure 1(b)). Gene Ontology (GO) is a commonly used bioinformatic tool that provides comprehensive information on gene function of individual genomic products based on defined features [39]. This analysis was performed to establish the role of enrichment in molecular functions (MF), biological processes (BP), and cellular components (CC) in the interaction networks of C/EBP*β*. The enrichment analysis showed that for biological processes in the C/EBP*β* system, most of the genes were enriched at “cellular response to oxidative stress.” The cellular component analysis showed that most of the genes were “nuclear chromatin.” The typical molecular functions were “glucocorticoid receptor binding” and “protein heterodimerization activity” (Figure 1(c)). Hence, these results indicated that oxidative stress can be an effective target for PD research and presented more possible research directions for in-depth investigation.

### 3.2. Baicalin Inhibits the Toxicity and Oxidative Stress in pLVX-Tet3G-*α*-Synuclein SH-SY5Y Cell Lines

**Figure 2:**
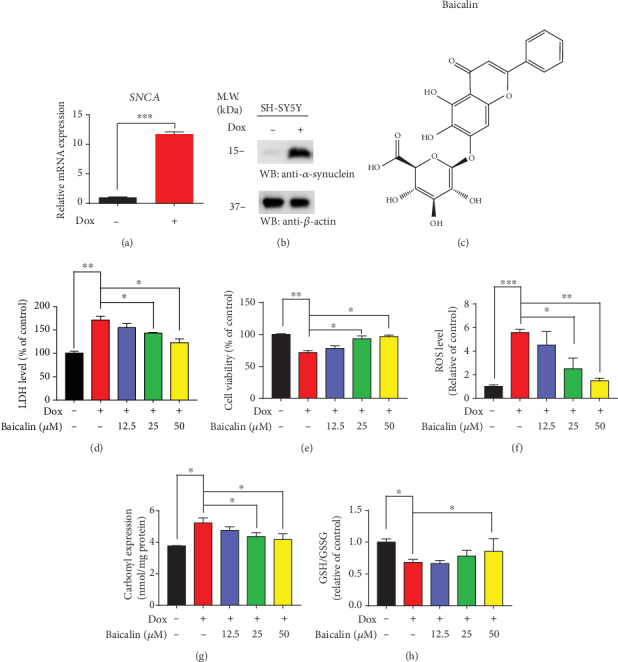
Baicalin inhibited toxicity and oxidative stress in pLVX-Tet3G-*α*-synuclein SH-SY5Y cell lines. (a) The mRNA level of *α*-synuclein in pLVX-Tet3G-*α*-synuclein SH-SY5Y stable cell line. (b) The protein level of *α*-synuclein in pLVX-Tet3G-*α*-synuclein SH-SY5Y stable cell line. (c) The chemical structure of Baicalin. (d) The LDH level of Baicalin. (e) Cell viability by MTT assay. (f) The ROS level, (g) carbonyl expression, and (h) GSH/GSSG level of Baicalin. The pLVX-Tet3G-*α*-synuclein SH-SY5Y cells were incubated with various concentrations (0–50 *μ*M) of Baicalin for 24 h. All data represented the mean and standard error of three independent experiments. ^∗^*p* < 0.05; ^∗∗^*p* < 0.01; ^∗∗∗^*p* < 0.001.

To examine the neurotoxicity of *α*-synuclein *in vitro*, the Doxycycline inducible pLVX-Tet3G-*α*-synuclein stable cell line was constructed, and the transfected efficiency was validated by mRNA and western blotting, as presented in Figures 2(a) and 2(b). To identify natural compounds capable of inhibiting the toxicity, we initiated a cell-based assay to screen chemicals extracted from Chinese herbal medicine [25]. The toxicity was evaluated by the release of lactate dehydrogenase (LDH). We found that 25 *μ*M or 50 *μ*M groups of Baicalin (Figure 2(c)) showed the significant ability (*p* = 0.036 and *p* = 0.02 individually) to protect cells from Dox-induced cell death (Figure 2(d)). Further study indicated that Baicalin exerted the protection effect on Dox-induced cells in a dose-dependent manner (Figure 2(e)). The quantitative oxidative stress level analysis showed that the 50 *μ*M Baicalin group decreased the ROS level (Figure 2(f)) (*p* = 0.007) and carbonyl expression (Figure 2(g)) (*p* = 0.039) while increasing the GSH/GSSH level (*p* = 0.048) (Figure 2(h)) compared to the Dox-induced group. Hence, Baicalin inhibited toxicity and balanced the redox homeostasis in pLVX-Tet3G-*α*-synuclein SH-SY5Y cell lines.

### 3.3. Baicalin Protects against Dox-Induced Mitochondrial Dysfunctions

**Figure 3:**
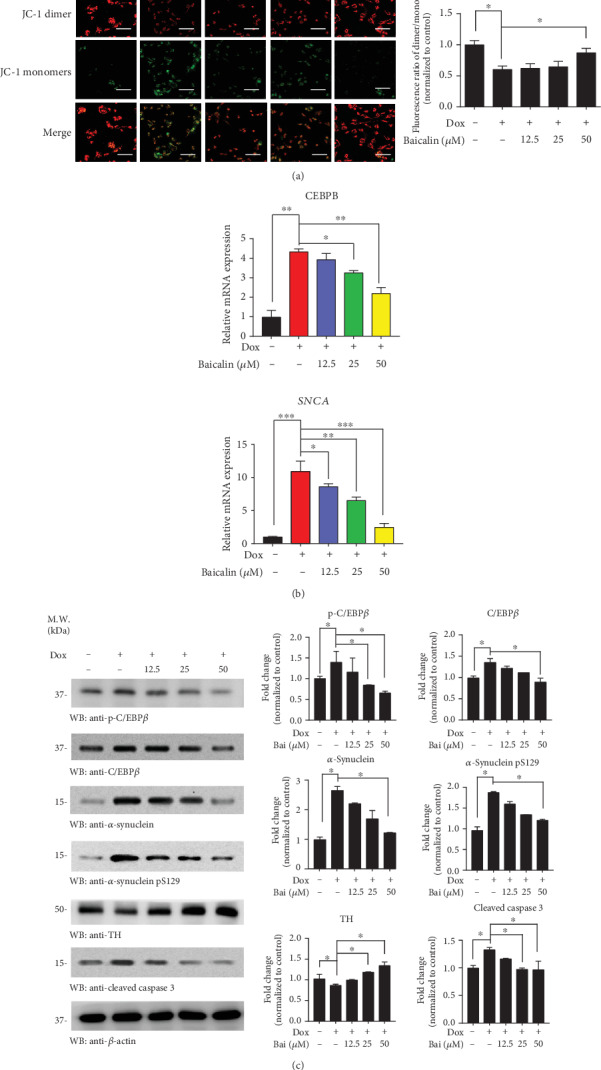
Baicalin protected Dox-induced mitochondrial dysfunction. (a) The representative image of pLVX-Tet3G-*α*-synuclein SH-SY5Y cell with an accumulation of JC-1 staining after incubation with various concentrations (0–50 *μ*M) of Baicalin for 24 h. Scale bars: 50 *μ*m. (b) The mRNA level of pLVX-Tet3G-*α*-synuclein SH-SY5Y cells treated with various concentrations (0–50 *μ*M) of Baicalin for 24 h. (c) Western blot analysis of pLVX-Tet3G-*α*-synuclein SH-SY5Y cell treated with various concentrations (0–50 *μ*M) of Baicalin for 24 h. All data represented the mean and standard error of three independent experiments. ^∗^*p* < 0.05; ^∗∗^*p* < 0.01; ^∗∗∗^*p* < 0.001.

To determine whether Baicalin possesses the preventive effects against mitochondrial dysfunctions, parameters of mitochondrial function were studied in pLVX-Tet3G-*α*-synuclein SH-SY5Y cell lines. Mitochondrial membrane potential (Δ*ψM*) is an important parameter of the mitochondrial function used as an indicator of cell health [40]. JC-1 is a dye that can selectively enter into mitochondria and reversibly change color from green to red as the membrane potential increases [41]. In Figure 3(a), Dox-induced JC-1 monomers were detected, which means that damaged or unhealthy cells were upregulated. On the other hand, increasing the concentration of Baicalin could induce JC-1 aggregates (healthy cells), especially at 50 *μ*M (*p* = 0.024). These results indicated that Baicalin could protect membrane potential from Dox-induced toxicity. To assess whether Baicalin regulates *CEBPB* and *SNCA* mRNA expression, we conducted quantitative RT-PCR (qRT-PCR) assays with pLVX-Tet3G-*α*-synuclein SH-SY5Y cells and found that *CEBPB* and *SNCA* were upregulated after DOX treatment and it can be reversed by Baicalin in a dose-dependent way (Figure 3(b)). Immunoblotting showed that TH proteins revealed a dose-dependent elevation while *p*-C/EBP*β*, C/EBP*β*, *α*-synuclein, and *α*-synuclein pS129 decreased in pLVX-Tet3G-*α*-synuclein SH-SY5Y cells upon Baicalin treatment. The results demonstrated that Baicalin could reverse Dox-induced *α*-synuclein aggregation. Interestingly, Dox-induced caspase-3 activation indicates that active oxidative stress triggers apoptosis. On the other hand, Baicalin reduced caspase-3 activation in a dose-dependent manner (Figure 3(c)).

### 3.4. Baicalin Protects Dopaminergic Neurons and Rescues Motor Dysfunction against MPTP-Induced Neurotoxicity *In Vivo*

**Figure 4:**
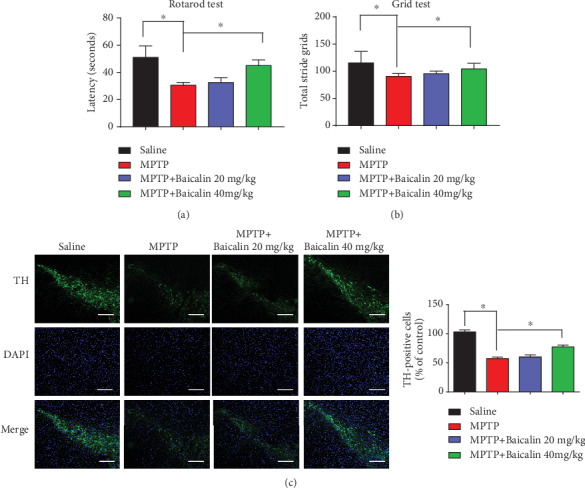
Baicalin protected dopaminergic neurons and rescues motor dysfunction against MPTP-induced neurotoxicity *in vivo*. (a) Rotarod tests and (b) grid tests were conducted by a blinded observer after two weeks of 20 mg/kg and 40 mg/kg Baicalin treatment. Data were the mean ± SEM (*n* = 8 per group). (c) TH staining of the substantia nigra (SN) of the above mice. Scale bars: 200 *μ*m. All data represented the mean and standard error of three independent experiments. ^∗^*p* < 0.05; ^∗∗^*p* < 0.01; ^∗∗∗^*p* < 0.001.

To investigate the *in vivo* roles of Baicalin, three-month-old C57BL mice (8 per group) were treated with MPTP (i.p., 30 mg/kg) or saline for 5 days. Motor behavioral tests showed that MPTP incurred significant motor disorder, which was ameliorated upon two weeks of 20 mg/kg and 40 mg/kg Baicalin treatment. Remarkably, in the 40 mg/kg Baicalin group, MPTP elicited in the rotarod test (*p* = 0.042) and grid test (*p* = 0.047) were significantly less severe than the control group (Figures 4(a) and 4(b)), supporting the fact that Baicalin was highly neuroprotective and prevented MPTP-elicited motor dysfunctions in mice. We also monitored dopaminergic neuron loss after Baicalin treatment. Immunofluorescence staining showed that dopaminergic neurons in SN were substantially diminished by MPTP as compared to the vehicle group. Again, Baicalin attenuated the loss of dopaminergic neurons (Figure 4(c)). The maximal neuroprotective effects occurred with the 40 mg/kg Baicalin group (*p* = 0.012).

### 3.5. Baicalin Regulates the Redox Balance in the MPTP Treatment Group

**Figure 5:**
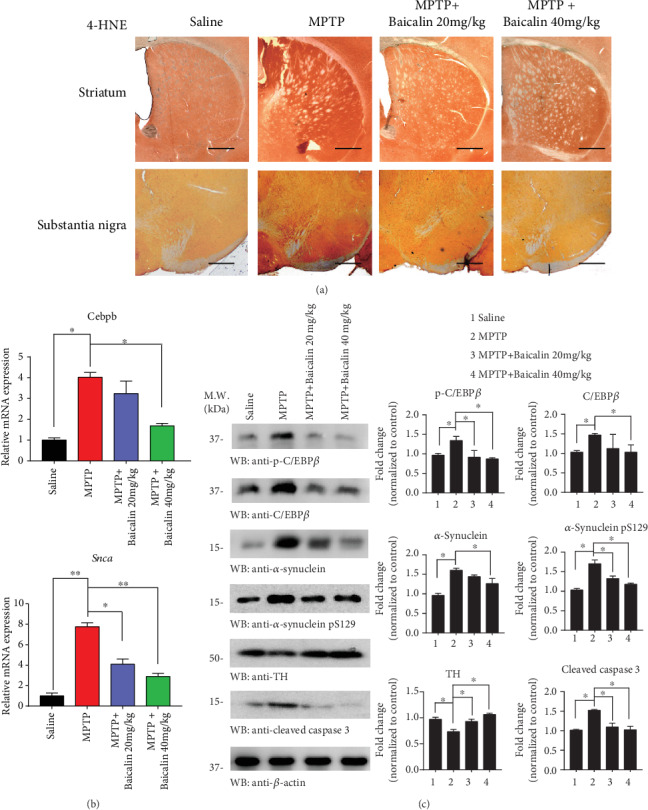
Baicalin protected dopaminergic neuron oxidative stress against MPTP treatment. (a) Immunohistochemical analysis for assessment of 4-HNE in the substantia nigra (SN) and striatum of saline, MPTP, MPTP+20 mg/kg Baicalin, and MPTP+40 mg/kg Baicalin groups. Scale bars: 500 *μ*m. (b) The *Cebpb* and *Snca* mRNA levels of represented groups. (c) SN lysates were probed with various indicated antibodies. The band densitometric data of WB. All data represented the mean and standard error of three independent experiments. ^∗^*p* < 0.05; ^∗∗^*p* < 0.01; ^∗∗∗^*p* < 0.001.

For manifesting the oxidative stress in Parkinson's disease, we employed 4-HNE staining, one of the most bioactive and studied lipid peroxidation biomarkers [42]. IHC analysis demonstrated that Baicalin group mice possessed the least oxidative stress among the experimental groups, provoked by MPTP treatment, in alignment with its prominent neuroprotective activity in the striatum and substantia nigra (Figure 5(a)). We observed that *Cebpb* (*p* = 0.021) and *Snca* (*p* = 0.0042) mRNA expression decreased after 40 mg/kg Baicalin treatment in SN compared to the MPTP group (Figure 5(b)). Immunoblotting analysis revealed the comparable protein levels expressed in the mouse brains. MPTP treatment elicited prominent dopaminergic neuronal TH loss, which was partially alleviated by Baicalin. As expected, *α*-synuclein pS129, *α*-synuclein, p-C/EBP*β*, and C/EBP*β* were strongly upregulated in the MPTP group after Baicalin treatment (Figure 5(c)). Hence, Baicalin possesses strong antioxidative activity, rescuing dopaminergic neurons from MPTP-induced cell death.

### 3.6. Baicalin Protects against Neuroinflammation and Oxidative Stress Triggered by MPTP Treatment

**Figure 6:**
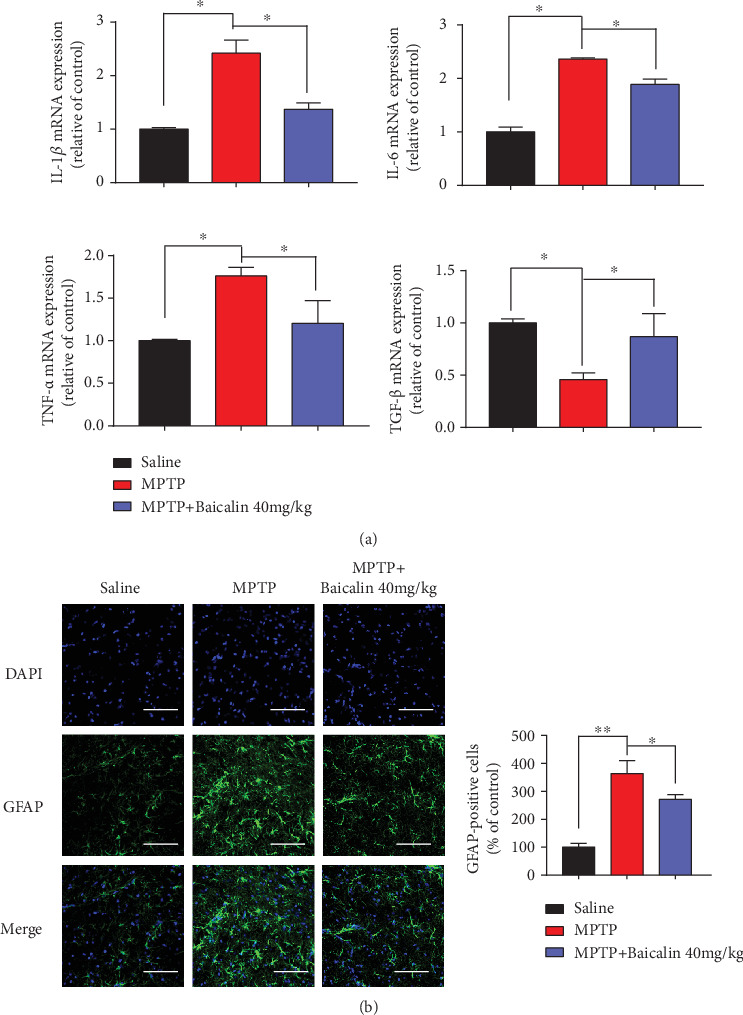
Baicalin protected against neuroinflammation and oxidative stress triggered by MPTP treatment. (a) IL-1*β*, IL-6, TNF-*α*, and TGF-*β* mRNA levels of SN measured by q-RTPCR in saline, MPTP, and MPTP+40 mg/kg Baicalin groups. (b) Immunofluorescence staining to detect the expression of the glial fibrillary acidic protein- (GFAP-) positive astrocyte (green) in saline, MPTP, and MPTP+40 mg/kg Baicalin groups. Scale bars: 100 *μ*m. All data represented the mean and standard error of three independent experiments. ^∗^*p* < 0.05; ^∗∗^*p* < 0.01; ^∗∗∗^*p* < 0.001.

Activated microglia and increased levels of inflammatory mediators are detected in the striatum of deceased PD patients [43, 44]; meanwhile, a large body of animal studies supports the contributory role of inflammation in dopaminergic cell loss [45]. Quantification of neuroinflammation revealed that IL-1*β*, IL-6, and TNF-*α* mRNA levels were decreased after Baicalin treatment, while the TGF-*β* mRNA level was increased (Figure 6(a)). Increased expression of GFAP is considered as markers of ROS production and the inflammatory process. In Figure 6(b), immunofluorescence staining of GFAP in the striatum showed a remarkably higher expression of GFAP-positive astrocytes in MPTP treatment when compared to the saline group (*p* = 0.0025). However, Baicalin decreased the GFAP-positive cells as well (*p* = 0.032). Again, our results indicated that Baicalin reduced oxidative stress in the MPTP treatment group.

## 4. Discussion

Over thousands of years, Chinese herbs have been used by the Chinese pharmacologists as treatment of diseases, including cancer [46], climacteric syndrome [47], schizophrenia [48], Alzheimer's disease [49], and Parkinson's disease [50]. Baicalin is one of the most important flavonoid compounds, which is mainly isolated from the root of Scutellaria baicalensis Georgi, which is an indispensable Chinese medicinal herb [51]. Besides, Baicalin does not display any significant toxicity to the mice at a dose even of 15 g/kg [52], which indicates its low toxicity, making it highly acceptable and safe for application in humans. However, one major limitation in the clinical application of Baicalin is their poor oral bioavailability and low aqueous solubility. There is increasing evidence that Baicalin plays an important role in various diseases, such as cardiovascular disease, depression, Alzheimer's disease, and Parkinson's disease. Baicalin alleviates cardiac dysfunction and myocardial remodeling in a chronic pressure overload mouse model [53]. In AD, Baicalin can reduce Alzheimer-like pathological changes and memory impairment caused by amyloid *β*1-42. Jin et al. reported that Baicalin can reduce cognitive impairment and protect neurons from microglia-mediated neuroinflammation TLR4/NF-*κ*B signaling [54]. In a recent study, Baicalin inhibited TLR4 expression through the PI3K/AKT/FoxO1 pathway and improved depression-like behavior [55]. Noticeably, Baicalein, which is the aglycone of Baicalin, inhibits *α*-synuclein fibrillation and disaggregates the preformed fibrils [56]. The medical mechanism of Baicalin inhibiting *α*-synuclein aggregates is still unclear. In our experiment, an *in vitro* experimental model, Baicalin (50 *μ*M) protected pLVX-Tet3G-*α*-synuclein SH-SY5Y cells against Dox-induced toxicity and also prevented the loss of cell viability (Figure 2(e)). Other researchers also found that lack of *α*-synuclein in mice is associated with reduced vulnerability to MPTP and reduced dopaminergic neuronal cell death [57]. The previous study also showed that abnormal posture, gait, and stiffness of the limbs are directly related to the loss of dopaminergic neurons, which is the root cause of PD [58]. Baicalin and deferoxamine can reduce iron accumulation in SN of Parkinson's disease rats [59]. In our results, we showed that Baicalin blocked *α*-synuclein expression and aggregation and protected dopaminergic neurons and rescues motor dysfunction against MPTP-induced neurotoxicity *in vivo* (Figure 4); presumably, Baicalin's therapeutic efficacy might partially result from its inhibition against *α*-synuclein.

Additionally, Baicalin may sustain redox homeostasis by protecting mitochondrial systems after treating with MPP+ [60]. Maintaining homeostasis is essential for preventing and curing disease [61]. Unbalanced homeostasis with the more oxidized environment, for example, higher oxidative stress, facilitated more cell death. In our research, Baicalin inactivated ROS (25 *μ*M and 50 *μ*M) production, carbonyl expression, and lactate dehydrogenase (LDH) level, meanwhile, alternated in GSH/GSSG ratio (Figures 2(d) and 2(f)–2(h)), facilitating a dramatically less oxidized environment for cell survival. Interestingly, Baicalein was effective in blocking the Dox-induced toxicity on the mitochondrial membrane potential (Figure 3(a)), resulting in increased ATP synthesis.

Currently, with the advent of next-generation sequencing technologies, RNA-Seq is gradually replacing microarrays for the detection of transcript expression profiling [62]. Although a lot of papers reported the RNA networks in PD, microarray methods were primarily employed [63, 64]. In this study, we used RNA-Seq to examine the global expression profiles of noncoding RNAs. Using RNA-Seq technology, we found that C/EBP*β* was upregulated in PD patient's blood samples (Figure 1(b)). Moreover, the target mRNAs of differentially expressed mRNAs were mostly involved in “glucocorticoid receptor binding,” “cellular response to oxidative stress,” and “protein localization to the mitochondria” (Figure 1(c)). These results were partly consistent with those of our data. Also, RNA-Seq data showed new findings in that regulation of “protein heterodimerization” and “nuclear chromatin” were involved in the pathogenesis of PD, which are interesting targets for further investigation.

Our previous work demonstrated that CEBP/*β*, which is the transcription factor of *α*-synuclein and MAO-B, mediates the pathogenesis of Parkinson's disease [65]. C/EBP*β* is one of the family members of transcription factors in the basic-leucine zipper (bZIP) class. In glial cells, C/EBP*β* regulates the proinflammatory program. Because of its role in neuroinflammation, C/EBP*β* is a potential target for the treatment of neurodegenerative disorders [66]. In our study, Baicalin repressed DOX-induced C/EBP*β* level and *α*-synuclein as indicated in mRNA and protein level (Figures 3(b) and 3(c)). In addition, it was demonstrated that Baicalin inhibited MPTP-triggered inflammatory mediators, including the classical proinflammatory triad of IL-1*β*, IL-6, and TNF-*α* (Figure 6(a)). Notably, Baicalin was involved in the regulation of proinflammatory gene expression in glial activation, especially GFAP expression, and played a key role in the reduction of neurotoxic effects by inactivating microglia (Figure 6(b)).

There may be some other limitations to our study that should be shortly mentioned: (1) Further studies are necessary to reveal how Baicalin regulates the inactivation of glial cells in PD models. (2) Although flavonoids are nonspecific inhibitors [67], our future research will focus on this basis and look for structural elements with high specificity and beneficial effects in PD treatment.

## 5. Conclusions

Our data strongly supported that the analysis of the gene expression profile may enable the identification of targets for PD diagnosis and treatment. The results confirmed that C/EBP*β* and oxidative stress played important roles in the progression of PD. Meanwhile, Baicalin functioned as an antioxidant for dictating the expression of C/EBP*β* and regulated oxidative stress in the pLVX-Tet3G-*α*-synuclein SH-SY5Y cell model and MPTP treatment mouse model, further indicating Baicalin's neuroprotective effect in neurotoxin-triggered Parkinson's disease.

## Data Availability

The data used to support the findings of this study have not been made available because the data also form part of an ongoing study.
